# Treatment recommendations within the leeway of clinical guidelines: A qualitative interview study on oncologists’ clinical deliberation

**DOI:** 10.1186/s12885-017-3783-6

**Published:** 2017-11-21

**Authors:** I. Otte, S. Salloch, A. Reinacher-Schick, J. Vollmann

**Affiliations:** 1 Institute for Medical Ethics and History of Medicine, Ruhr University Bochum, Markstr. 258a, D-44795 Bochum, Germany; 2 Institute for Ethics and History of Medicine, University Medicine Greifswald, Ellernholzstr. 1-2, D-17487 Greifswald, Germany; 3 Department for Hematology, Oncology and Palliative Care, St. Josef-Hospital, Ruhr-University Bochum, Gudrunstr. 56, D-44791 Bochum, Germany

**Keywords:** Pancreatic carcinoma, Treatment recommendation, Clinical deliberation, Clinical guidelines, Clinical decision-making, Qualitative research

## Abstract

**Background:**

Recommending the optimal treatment for an individual patient requires a well-balanced consideration of various medical, social and ethical factors. The interplay of these factors, interpretation of the patient’s situation and understanding of the existing clinical guidelines can lead to divergent therapy recommendations, depending on the attending physician. Gaining a better understanding of the individual process of medical decision-making and the differences occurring will support the delivery of optimal individualized care within the clinical setting.

**Methods:**

A case vignette of a 64-year-old patient with locally advanced pancreatic adenocarcinoma was discussed with oncologists in 14 qualitative, semi-structured interviews at two academic institutions. Relevant factors that emerged were ranked by the participants using the Q card sorting method. Qualitative data analysis and descriptive statistics were performed.

**Results:**

Oncologists recommend different therapeutic approaches within the leeway of the relevant clinical guidelines. One group of participants endorses a rather aggressive and potentially curative approach with a combination chemotherapy following the FOLFIRINOX protocol to provide the patient with the best chances of resectability. The second group suggests a milder chemotherapy approach with gemcitabine, highlighting the palliative approach and the patient’s quality of life. Clinical guidelines are generally seen as an important point of reference, but are complicated to apply in highly individual cases.

**Conclusion:**

The physician’s individual assessment of factors, such as biological age, general condition or prognosis, plays a decisive role in treatment recommendations, particularly in those cases which are not fully covered by guidelines. Judgment and discretion remain crucial in clinical decision-making and cannot and should not be fully ruled out by evidence-based guidelines. Therefore, a more comprehensive reflection on the interaction between evidence-based medicine and the physician’s estimation of each individual case is desirable. Knowledge of existing barriers can enhance the implementation of guidelines, for example, through medical education.

**Electronic supplementary material:**

The online version of this article (10.1186/s12885-017-3783-6) contains supplementary material, which is available to authorized users.

## Background

Choosing and recommending a suitable treatment option for an oncological patient is a complex task which requires a well-balanced consideration of various factors. A physician’s professional knowledge and experience, the newest scientific findings and clinical guidelines, are among these factors. However, institutional, social, legal and ethical factors also influence the final treatment recommendation.

Empirical evidence suggests that a physician’s personal beliefs play a key role in the development of an initial hypothesis, the ways in which they search for evidence and the mechanisms involved in deriving a final treatment plan [1]. The use of clinical guidelines by physicians was also shown to be linked to several factors, such as the evidence incorporated in these guidelines, the physician’s expertise, their relationship to the patient, and the patient’s medical and social situation [2–4]. Research has further shown that the decision-making process and treatment selection can also be influenced by patient-sided factors, such as a patient’s demographics or socioeconomic status [5–8].

While research has identified the influence of these factors, the way in which physicians weigh and prioritize them concretely during decision-making processes has not yet been well documented. The reasons for preferring one treatment over the other remain especially unclear [9]. Differences in treatment recommendations are, however, of great practical importance, as patients may receive divergent treatment offers depending on their choice of attending physician. This situation is aggravating in situations with multiple treatment options which are highly preference-sensitive [10], or in situations in which not only the treatment offer, but also the intended therapy goal may vary. Analyzing the individual process of weighing one factor against another is, therefore, key to a better understanding of differences in medical decision-making. Gaining more knowledge and insight into this process could support the delivery of optimal individualized care within the clinical setting.

Decisions about the intensity of oncologic care are highly influenced by national contexts and health care systems. Health care coverage in Germany is universal and mandatory for all citizens. Approximately 85% of the population is covered by the statutory health insurance (SHI), which covers all medical services and procedures that are adequate, practicable and cost-effective. Private health insurance covers a benefit package similar to SHI, but can also include additional options. Therefore, the German population, with rare exemptions, receives funding for all cancer treatments which are judged as beneficial from a medical and efficiency perspective.

## Methods

We conducted a qualitative interview study on oncologists’ treatment recommendations in individual cases to gain a better understanding of the process of clinical deliberation. Following approval from the ethics committee of the Medical Faculty of the Ruhr-University Bochum (Reg. No. 5025–14), 14 semi-structured qualitative interviews were conducted, each of which included the same case vignette (see Table 1).

**Table 1:** Case vignette

Patient demographics	M, 64 yrs.; married
Status praesens	Nausea and pain in the upper abdomen, back pain, unintended weight loss of 6 kg, condition stable, but generally restricted.
Past medical history (PMH)	Three-vessel CAD; s/p CABG occasionally AP on exertion-controlled arterial hypertension, COPD GOLD II, under medication.
Family history (FH)	Unknown
Social History (SH)	Smoker (60 pack-years), lives at home with his wife who supports him with domestic activities; he formerly worked as a butcher, now unfit for work.
Allergies (ALL)	None
Physical examination (PE) and test results	- Slightly diminished general condition, BMI 27, 3, 140/95, heart and lungs: NAD, neurology: NAD - Endoscopic ultrasound: mass located in the pancreatic head, inhomogeneous - MRI scan abdomen: 3.5 cm mass in processus uncinatus, multiple peripancreatic lymph nodes - MR angiography: primary cancer not resectable: full vessel involvement and infiltration of SMA - Histology (endosonography): high-grade pancreatic ductal adenocarcinoma, no metastases detected - LAB: Hemoglobin 13.2 g/dl; Leukocyte 5200/μl; Thrombozyten 150,000/μl Creatinin: 1.2 mg/dl Bilirubin: 1.1 mg/dl Amylase: 100 U/l Lipase: 55 U/l CA19–9 (carbohydrate antigen 19.9): 1350 U/ml
Diagnosis	Locally advanced, pancreatic ductal adenocarcinoma cT4N1M0 (UICC stage III)

The case vignette was discussed by using the card sorting method (Q methodology), which is suitable for semi-quantitative factor analysis [11]. While rating the different factor cards (on a scale with 5 being the highest influence on medical decision-making and 0 being no influence at all) regarding the case vignette, the participants were asked to explain the rationale underlying their ratings verbally. Consequently, the card sorting was also used as a discussion prompter to start an in-depth narration which enabled us to elaborate further on the underlying attitudes and values behind the participants’ treatment choices.

The focus of the interviews was on thematic aspects, such as intuition, evidence-based medicine (EBM), work experience, clinical guidelines and their perceived importance for medical decision-making (Table 2). Based on the literature review, factor cards were developed to represent four categories of possible influences on medical decision-making: colleagues, patient and family, EBM-related factors (guidelines, scientific literature) and personal values. The factor cards were pretested during the first interviews, giving the interviewees the opportunity to add relevant factors to the card set. Together with the qualitative semi-structured interview part (see Additional file 1 for the interview guide), we reached a level of detail which is usually not obtainable by using only a purely traditional statistical technique.

**Table 2:** Factors rated regarding their influence on physicians’ decision-making

Factor cards	Rated importance (5 > 0) mean:
Patient’s wishes and preferences	4.86
Clinical guidelines	4.29
Patient’s general condition	4.2
Medical literature	4.14
Supervisors	4.07
Medical co-workers	3.93
Nurses	3.77
Patient’s age	3.46
Professional experience	3.43
Support from relatives/social environment	3.36
Intuition	3.21
Physician’s own values	3.20
Institutional guidelines	3.07
Psycho-oncologist	2.85
Wishes and interests of patient’s relatives	2.57

5 being the greatest influence and 0 no influence at all

A purposive sample was drawn from three departments of two German university hospitals, both of which are comprehensive cancer centers for pancreatic malignancies. The main criterion for participants being included in the study was their current employment in the unit of medical oncology. Variation was sought regarding gender and professional experience in the treatment of oncological patients.

### Analysis

Interviews were transcribed verbatim. All transcripts were read and reread to ensure familiarity with the data. The coding process followed Mayring’s nine steps of content analysis: a) The relevant data was defined; b) the context of the appearance of the data registered; c) a formal characterization of the data material described; d) the course of analysis specified; e) a theory-lead differentiation checked; f) the technique of analysis defined (summarization, explication, structuring); g) the unit of analysis defined; h) the data material analyzed; and, finally, i) the data material interpreted [12]. The data was repeatedly coded, moving from concrete passages to abstract levels of coding, deriving themes from the data and searching for repeated concepts. All findings were critically tested and discussed in team meetings. Any disagreements were resolved by discussion. Since the coding system remained the same for the last interviews and the findings did not add something significantly new to the interviews conducted previously, we concluded that we had reached saturation.

## Results

The sample consisted of eight male and six female oncologists aged between 30 and 53 years (median age: 38 years), with a median clinical experience of 9 years. Six participants were still in their residency. The participants rated different factors which influenced their decision-making:

The participating oncologists recommended different therapeutic approaches in the second part of the interviews, while also providing insights into their clinical deliberation and decision-making routine. Whereas a minority of the participants assumed that there was still a chance to reach operability in the case vignette, most thought that reaching operability was unlikely and a palliative approach was the better option for the patient. The analysis revealed that the participants’ recommendations could be divided into two major groups in which the physicians recommended either an aggressive type of chemotherapy with the FOLFIRINOX regimen or the more moderate form of a monotherapy with gemcitabine. Two participants recommended approaches diverging from these schemes: One physician recommended a merely supportive therapy and the other physician suggested initial laparoscopic surgery for diagnostic purposes.

### Treatment recommendation A: Combination chemotherapy with Oxaliplatin, Leucovorin, Irinotecan and 5-FU (FOLFIRINOX protocol)

Physicians in this group recommended an aggressive type of cytostatic therapy with FOLFIRINOX, because they believe it offers a better probability of shrinking the primary cancer to achieve resectability.

*IP 3: “I would recommend chemotherapy with FOLFIRINOX, because I can see that the patient could reach resectability with this chemotherapy using FOLFIRINOX.”*

Their hope was that by shrinking the primary cancer, the chances for a surgical removal would increase, resulting in a possibly curative approach.

*IP 14: “And he is not bedridden, so when we start with chemotherapy following the FOLFIRINOX regimen, it could be that the pancreatic tumor shrinks in a certain way, which means, that probably in the future, it could become operable, so that surgery could be an option, but, in any case, you would start with an intensive, strong chemotherapy following the FOLFIRINOX regimen.”*

The participants from this group, furthermore, think that the patient is relatively young and, therefore, in a sufficiently good condition to undergo this rather aggressive regimen.

*IP9: “So the patient is of a relatively young age and has a severe carcinoma with a very poor prognosis. That means I would offer him chemotherapy with FOLFIRINOX in view of a possible resectability of the tumor.”*

### Treatment recommendation B: Palliative monochemotherapy with gemcitabine

Physicians who recommended a mild type of chemotherapy with gemcitabine often aim at a palliative approach, with a stronger focus on quality of life rather than on resectability, since they assess the chances of reducing the tumor mass as rather low. They further believe that the general condition of the patient is not good enough to endure an aggressive form of chemotherapy and, therefore, select a regimen that they think would be better tolerated by the patient:

*IP 4: “So (..) he is in a moderate condition; there is this FOLFIRINOX which you could give, but many do not tolerate it well. When I look at the case, he has COPD, he has a cardiac disease. (…) it is questionable if he could tolerate it.”*

While acknowledging that there are potentially curative approaches, participants of this group rather suggest going with a milder form of chemotherapy to prolong the time of survival, while still offering the patient a good quality of life:

*IP 5: “I’d say I would decide between a system therapy with gemcitabine mono or, what we could also think about is a combination of gemcitabine, maybe he can tolerate it in reduced doses. It is a bit difficult, because his carcinoma is theoretically shrinkable, but it is cT4, and the chances that this ends in resectability are very low, especially since he is probably limited in his ability to tolerate therapy due to his pre-existing condition.”*

In contrast to the previous group, the oncologists in the second group thought the patient was “not the youngest” and, therefore, probably not capable of undergoing a more aggressive protocol.

*IP 12: “One therapy option would be palliative chemotherapy with gemcitabine, which could, in the case of pancreatic carcinomas, be at least effective regarding the total time of overall survival. (…) He is not the youngest, so that I fear this patient would not be offered FOLFIRINOX.”*

### Dealing with guidelines

While discussing and deciding their therapy recommendations, the physicians mentioned guidelines as an important factor and a framework on which they could orientate their decision-making. Clinical guidelines were the second highest factor influencing the physicians’ decision-making in the card-sorting part of the interview. The participants were asked to explain how they use clinical guidelines in their decision-making process and what possible difficulties they could face in guideline implementation, to gain more insight into the role and the importance of guidelines. One of the difficulties named repeatedly was that guidelines often refer to a prototype of patient who might differ from the actual person the physician is treating:

*IP12: “Guidelines often refer to a standard patient and we do not deal with a standard patient in the concrete situation. So we have to adapt the guidelines to the concrete patient we are treating.”*

This can be particularly difficult when the guidelines are based on studies which included significantly younger patients with a better general condition:

I*P7: “Guidelines, for example, are based on some studies that were conducted on young and rather healthy patients and these results aren’t always transferrable to older patients or patients in a severe state, so we have to adapt the guidelines to match our cases. Typical situations would be the FOLFIRINOX regimen, which causes the patient, Mr. M, from the case vignette, for example, a lot of side effects, but the studies that were conducted on younger and healthier patients, well, these patients could deal with the side effects much better and experienced fewer of them.”*

Furthermore, the physicians stated that they face situations in which guidelines do not provide clear guidance, for example, in cases of recurring disease or therapy failure:

*IP4: “There are cases in which guidelines do not apply, where you have a relapse and there is no third-line therapy or fourth-line therapy available, right, when you have already tried so much (..) Or in cases where the first-line therapy is not well tolerated and in which we have to try something different. Something weaker which might not have as good an outcome, but which does not kill or unnecessarily weaken the patient. It depends on the general condition of the patient, but there are also cases that are so rare that there hasn’t been any research yet.”*

Sometimes the implementation was also found to be difficult because the guidelines are presented in an oversimplified way:

*IP3: “Well, in the end, guidelines are very generic, which means they address certain age groups or patients that have benefited from a certain type of chemotherapy in a certain way. And this does not cover all the different factors (…), like patient preferences or social environment, sometimes the guidelines cover the age, but overall it is all very simplified.”*

The interview analysis generally demonstrates that the concrete treatment recommendation physicians make to an oncologic patient depends highly on their subjective estimation of the patient’s biological age and prognosis. Clinical guidelines are seen as an important point of reference, but cease being helpful in highly individual cases.

## Discussion

German oncologists generally adhere to the German S3 guidelines, which are consensus guidelines based on an elaborate formal evidence assessment where official evidence tables are published together with the guideline. International guidelines, such as the ASCO or ESMO guideline, are also taken into consideration in the process of the German guideline development, with the goal of an international harmonization of the guidelines. The German guideline for pancreatic cancer [13], which was published in 2013, is presently being revised in an elaborate process. In the meantime, many German oncologists refer to international guidelines, such as the NCCN guidelines, for more recent recommendations [14].

The case vignette in our study referred to a 64-year-old patient with locally advanced pancreatic adenocarcinoma (LAPC) and a supposed Eastern Cooperative Oncology Group (ECOG) performance status between 1 and 2. According to US guidelines, patients who have LAPC and an ECOG of 0 or 1 together with a favorable comorbidity profile and a support system for aggressive medical therapy should receive an initial systemic therapy with combination regimens [4, 5]. However, there is no clear evidence to support one regimen over another, and it is recommended that physicians offer therapy on the basis of extrapolation from data derived from studies in the metastatic setting [5]. Newer regimens such as fluorouracil, leucovorin, irinotecan and oxaliplatin, and gemcitabine plus nanoparticle albumin-bound paclitaxel have not been evaluated in randomized controlled trials (RCTs) involving LAPC, but these combination regimens are being frequently used by clinicians and, thus, may be recommended for patients with LAPC with good performance status (ECOG 0 and 1) [14]. For patients with LAPC, regimens with gemcitabine only, or gemcitabine plus capecitabine (GEMCAP) alone or in combination are potentially better tolerated and might be considered a better option in patients with a borderline ECOG or based on patient preference. The German S3 guideline on exocrine pancreatic adenocarcinoma more generally suggests that patients with a performance status from 0 to 2 should receive palliative chemotherapy [3].

This spectrum of therapeutic options which could be applied in adhering to guidelines is mirrored in the study participants’ answers regarding the case vignette. The qualitative interviews also give some indication about when oncologists tend to suggest a more or less aggressive treatment. The most important factors that influence the choice of an aggressive or less aggressive treatment are the general condition of the patient, often in combination with their age. While participants who suggest the more aggressive option judge the patient to be in an overall good general condition and rather young, oncologists who suggest a milder version believe that the patient is “not the youngest” and that his preexisting medical conditions would limit his tolerance and ability to undergo this regimen. These findings are in accordance with previous research, which demonstrates that younger patients are more likely to receive aggressive care near the end of life [15, 16]. The present interview study, however, sheds light on the fact that the factor of the “patient’s age” depends highly on the attending physician’s estimation. The study also demonstrates that the diverging clinical judgement of the probability of reaching resectability has an immense impact on the decision-making and the final treatment recommendation. Oncologists who assessed the chance to reach operability in the case vignette presented as rather low, focused on time-prolonging measures, while those who thought that operability was attainable, suggested the aggressive protocol with a higher chance of resectability and a more severe set of side effects. Assessment of resectability is, thus, identified as another factor which influences treatment aggressiveness in addition to other, known, factors, such as comorbidity [17] and socioeconomic variables [18].

Participants also explained their difficulties with the implementation of clinical guidelines. While some of the difficulties named by the oncologists were already known to exist for general practitioners (GPs), such as differences between actual patients and the research population or contradictive patient preferences [4, 19], our study adds insight to the implementation process of guidelines in the oncological setting, as well as new barriers that are related to it. Whereas Carlsen and Norheim showed that GPs find clinical guidelines to be too detailed [3], the oncologists in our study often found them too generic and, therefore, not applicable to their patients. They said it was particularly difficult to use guidelines when they were based on research that focus on younger and healthier patients, making it difficult to predict how patients in a more severe state would respond to such therapy or cope with its side effects. The absence of clinical guidelines in cases of relapse or third- or fourth-line therapy was also said to be problematic.

## Conclusions

In our study, the physician’s individual assessment of factors, such as biological age, general condition or prognosis, plays a decisive role in treatment recommendations, particularly in those cases which are not fully covered by guidelines. Physician’s judgment and discretion remain crucial in clinical decision-making and cannot be fully ruled out by evidence-based guidelines. Therefore, a more comprehensive reflection on the interaction between evidence-based guidelines and the physician’s assessment of each individual case are desirable, since knowledge of existing local, regional or international peculiarities or ethical barriers can assist the enhancement of the implementation of guidelines.

## Additional file

Additional file 1: Interview guide (PDF): This file contains the set of interview questions which has been used for the semi-structured interview part of this study. (PDF 110 kb)

## Abbreviations

ALL: Allergies
AP: Angina pectoris
BMI: Body mass index
CA19–9: Carbohydrate antigen 19.9
CABG: Coronary artery bypass grafting
CAD: Coronary artery disease
COPD: Chronic obstructive pulmonary disease
EBM: Evidence-based medicine
ECOG: Eastern Cooperative Oncology Group
FH: Family history
FOLFIRINOX: Combination chemotherapy protocol with oxaliplatin, irinotecan and 5-FU
GEMCAP: Combination chemotherapy protocol with gemcitabine plus capecitabine
GOLD: Global Initiative for Chronic Obstructive Lung Disease
GPs: General practitioners
HPI: History of present illness
LAPC: Locally advanced pancreatic adenocarcinoma
MRI: Magnetic resonance imaging
NAD: Nothing abnormal detected
PE: Physical examination
PMH: Past medical history
SH: Social history
SHI: Statutory health insurance
UICC: Union for International Cancer Control
