# Supplementary material for: Treatment recommendations within the leeway of clinical guidelines: A qualitative interview study on oncologists’ clinical deliberation
Source: BMC Cancer. 2017 Nov 21;17:780. doi: 10.1186/s12885-017-3783-6 (PMC5699200; doi:10.1186/s12885-017-3783-6)
Supplement: Additional file 1: — Interview guide (PDF): This file contains the set of interview questions which has been used for the semi-structured interview part of this study. (PDF 110 kb) [file 12885_2017_3783_MOESM1_ESM.pdf]

**Current Situation**	
Could you please start by describing your role and tasks in this clinic?	
**Case Vignette**	
[see separate document]	
**Case Discussion**	
Do you have any questions regarding this case?<br><br>Which therapy would you recommend to the patient?<br><br>Are there any alternatives to this therapy?	Understanding<br><br>Task<br><br>Therapeutic alternatives
**Card Sorting**	
I will now give you cards with words and we ask you to rate, which relevance these factors have for your decision.<br><br>I also give you a scale reaching from 0 to 5 whereas 5 means most important and 0 means completely irrelevant. We ask you to rate the factors on this scale. Multiple placements are possible, for example more than one card can be given the value 4. If a factor does not apply at all to your work, please rate it as „not applicable".<br><br>Are there cases where you would change this order, so weigh the factors differently?<br><br>Does it matter, from your perspective, *where* the patient is treated?	Cards<br><br>Task<br><br>Other cases<br><br>Influence clinic / place
**Interview Questions**	
Would you like to add something to the case vignette? (contentwise, formally, emotionally)	Comments to vignette

Did you find the case difficult to decide?<br><br>*If yes:* Could you describe another therapeutic situation which you think is difficult to decide?<br><br>*If no:* What would be a therapeutic situation which is difficult to decide?	„Difficult“ situations
We have talked about the relevance of study results for your decision-making. How do you get informed about these results (source) and how do you proceed in this?	Source of information
What does Evidence-Based Medicine mean for you?	EBM
What is your experience in applying clinical guidelines to your patients?<br><br>Do you experience any discrepancies between your own clinical experience, your intuition and clinical guidelines?	Experience clinical guidelines<br><br>Clinical experience
Assume you were the mentor of younger colleagues: What would you advise them to do in important therapeutic decisions? What should they refrain from? What would you wish them?<br><br>What has changed in your own clinical practice regarding your decision making?	Advice to young colleagues<br><br>Development of decision making
